# Therapeutic potential of natural medicines in diabetic kidney disease: restoring lipid homeostasis via lipophagy modulation

**DOI:** 10.3389/fphar.2025.1665339

**Published:** 2025-09-19

**Authors:** Junwei Gao, Yunzhou Wu, Xudong Cai, Hailing Zhao, Jie Xing

**Affiliations:** ^1^ Department of Nephrology, Ningbo Municipal Hospital of Traditional Chinese Medicine, Affiliated Hospital of Zhejiang Chinese Medical University, Ningbo, China; ^2^ Beijing Key Lab for Immune-Mediated Inflammatory Diseases, China-Japan Friendship Hospital, Beijing, China

**Keywords:** natural medicines, lipophagy, diabetic kidney disease, autophagy, renal lipidmetabolism, lipid homeostasis

## Abstract

Diabetic kidney disease (DKD), one of the most prevalent microvascular complications of diabetes mellitus, is characterized by a complex pathogenesis in which lipid metabolism dysregulation plays a central role. Increasing evidence indicates impaired lipophagy, a selective autophagic process responsible for degrading lipid droplets, contributes substantially to renal lipid accumulation and subsequent kidney injury in DKD. Natural medicines, leveraging their multi-target and multi-pathway regulatory properties, exert considerable therapeutic potential through modulation of lipophagy and restoration of lipid homeostasis. This review synthesizes current studies on the efficacy of natural medicines in enhancing renal lipophagy and attenuating lipid-mediated kidney injury in DKD. We systematically analyze major classes of natural medicines, including flavonoids, polyphenols, terpenoids, alkaloids, and polysaccharides, and discuss their mechanisms of action through key signaling pathways such as AMPK/mTOR, PPARα/γ, and SIRT1/FoxO1. These natural medicines effectively reduce renal lipid accumulation, mitigate oxidative stress and inflammation, and alleviate pathological damage in various DKD models. Their pleiotropic effects suggest promising therapeutic avenues for DKD through the restoration of lipophagic flux and lipid homeostasis. Nonetheless, significant challenges remain, including incomplete elucidation of precise molecular mechanisms and a scarcity of robust clinical validation. Future research must prioritize the rigorous identification of natural medicines, detailed mechanistic exploration, and well-designed clinical trials to translate the potential of natural medicine-mediated lipophagy regulation into effective therapeutic strategies for DKD.

## 1 Introduction

Diabetic kidney disease (DKD), a prevalent microvascular complication of diabetes mellitus, demonstrates a significant association with the progression of end-stage renal disease (Liu et al., 2024; Wu et al., 2023). Its pathogenesis encompasses multifactorial biological processes, including dysregulated glucose and lipid metabolism, hemodynamic alterations, oxidative stress, inflammatory responses, and fibrotic remodeling (Chen et al., 2023; Ma et al., 2023; Tian et al., 2024; Wang et al., 2023; Zhou et al., 2023). Lipid metabolic dysregulation is a critical contributor in DKD, as excessive lipid deposition in renal tissues disrupts glomerular filtration barrier integrity and accelerates kidney injury (Wang et al., 2024). Although pharmacotherapies such as statins have been widely used to correct dyslipidemia, their long-term application may paradoxically enhance renal lipid uptake and exacerbate ectopic lipid accumulation (Huang et al., 2023), highlighting the urgent clinical need for more effective interventions targeting lipid homeostasis. Lipophagy, a selective autophagic process that degrades intracellular lipid droplets to maintain lipid homeostasis, demonstrates functional impairment in DKD. Relevant studies have shown that lipophagy impairment contributes to lipid metabolic disorders, ultimately promoting renal fibrosis and accelerating DKD progression (Liu et al., 2023; Yang et al., 2023). Therefore, restoring lipophagy represents a promising therapeutic strategy to rebalance lipid metabolism and ameliorate renal injury in DKD.

**FIGURE 1:**
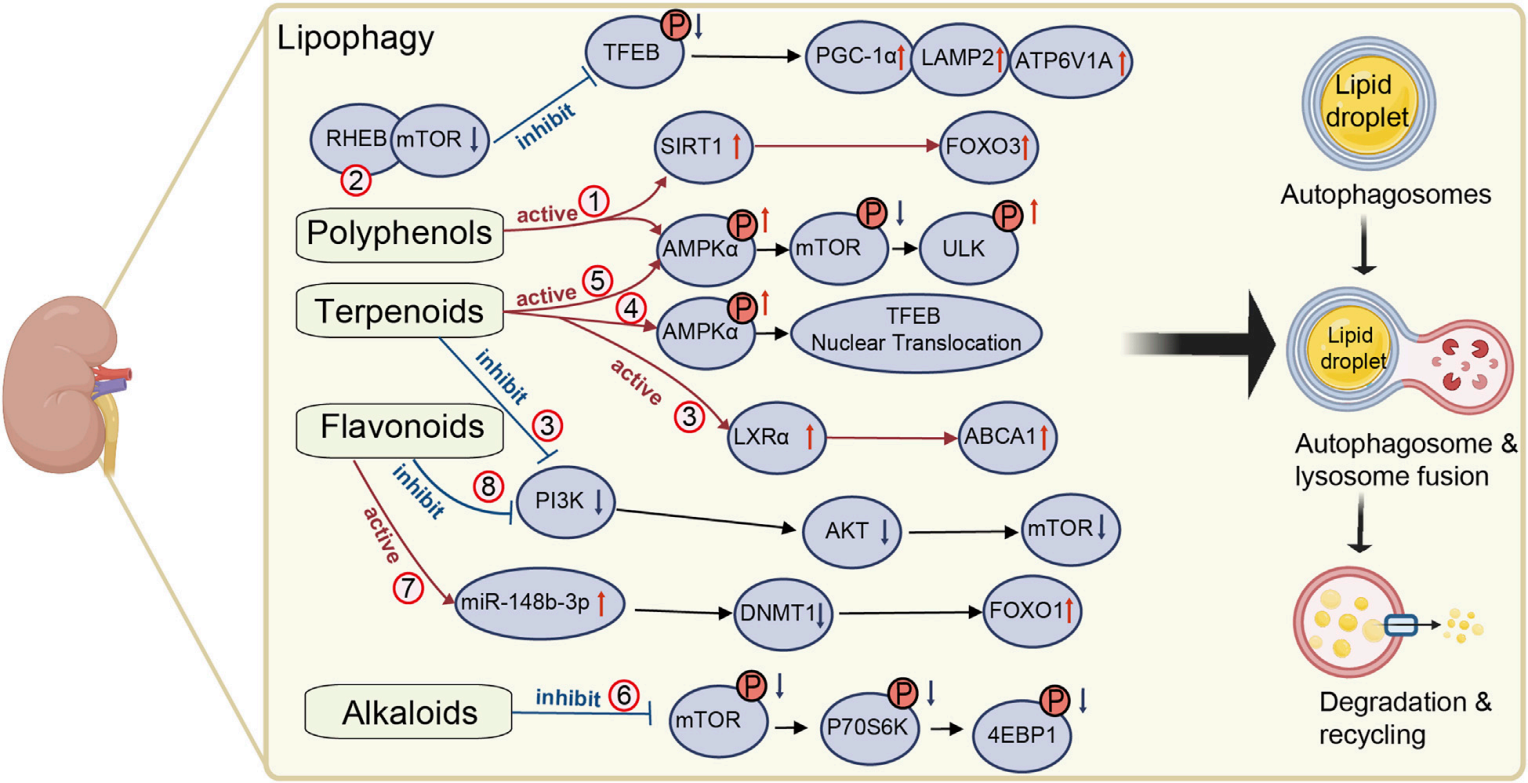
Mechanisms of natural medicines in promoting renal lipophagy. The mechanism of natural medicines promoting renal lipophagy is shown in Figure 1. It briefly describes the mechanisms underlying renal lipophagy and the molecular pathways by which natural medicines exert its beneficial effects. The red circles represent different natural medicines in the relevant regulatory pathways. (1) resveratrol, (2) paeonol, (3) celastrol, (4) morroniside, (5) geniposide, (6) berberine, (7) diosgenin, (8) calycosin. This figure was created using BioRender (https://biorender.com).

Natural medicines, characterized by their multi-component and multi-target properties, have shown unique advantages in modulating lipid metabolism and alleviating renal injury in DKD (Chung et al., 2023; Zhu et al., 2023). Notably, increasing evidence indicates that certain natural medicines can enhance lipophagy activity in kidney tissues, thereby improving lipid turnover and preventing ectopic lipid deposition. Elucidating the mechanistic relationship between lipophagy regulation and natural medicine interventions may provide novel insights for clinical management and future drug development. The mechanisms by which natural medicines promote lipophagy in the kidneys are detailed in Table 1 and Figure 1.

**TABLE 1:** Comparative analysis of natural medicines promoting renal lipophagy.

Chemical class	Natural medicines	*In Vivo/In Vitro*	Model	Signaling pathways or targets	Functional outcome	References	Strength of evidence
Polyphenols	Resveratrol	*In Vivo* and *In Vitro*	HK-2 cells Streptozotocin (STZ)-induced diabetic rats	SIRT1/FoxO3 Pathway (SIRT1↑, FoxO3↑, PGC-1α↑) AMPKα/mTOR/ULK1 Pathway (AMPKα↑, mTOR↓, ULK1↑)	Improves renal lipid accumulation and damage; Attenuates insulin resistance	Claudia, et al., 2022; Yong-Qi and Youjia (2020)	Strong
Polyphenols	Paeonol	*In Vivo* and *In Vitro*	glucolipotoxicity-treated HK-2 cells high-fat diet (HFD)+STZ-induced diabetic mice	RHEB/mTOR/TFEB Pathway (binds to RHEB, mTOR↓, TFEB↑, PGC-1α↑)	Reduces renal tubular lipid accumulation; Improves renal function	Ai, et al. (2025)	Strong
Terpenoids	Celastrol	*In Vivo* and *In Vitro*	HFD + STZ-induced diabetic rats; clear cell renal cell carcinoma cell; Nude mice with ccRCC (786-O) xenografts fed a HFD	PI3K/AKT/mTOR Pathway (PI3K/AKT↓, mTOR↓) LXRα/ABCA1 Pathway (LXRα↑, ABCA1↑)	Attenuates podocyte injury Improves renal function Reduces renal lipid accumulation Suppresses kidney tumor proliferation, migration, and growth	Chan-juan, et al., 2020; Nie, et al., 2020	Strong
Terpenoids	Morroniside	*In Vivo* and *In Vitro*	palmitic acid-induced HK-2 cell High-fat/high-fructose diet-fed C57BL/6 mice	AMPKα/TFEB Pathway (AMPKα↑, TFEB↑)	Improves renal function; reduces renal lipid accumulation; suppresses inflammation	Zhang C. et al. (2025)	Strong
Terpenoids	Geniposide	*In Vivo*	Unilateral nephrectomy + HFD + STZ-induced DKD mice	AMPK/ULK1 Pathway (AMPKα↑, ULK1↑)	Improves renal function	Theodomir, et al. (2021)	Moderate
Alkaloids	Berberine	*In Vitro*	high glucose-induced mouse podocytes	mTOR/P70S6K/4EBP1 Pathway (mTOR/P70S6K/4EBP1 phosphorylation↓)	Attenuates HG-induced podocyte apoptosis	Li, et al. (2020)	Preliminary
Flavonoids	Diosgenin	*In Vitro*	palmitic acid and high glucose-induced HK-2 cell	miR-148b-3p/DNMT1/FOXO1 Pathway (miR-148b-3p↑, DNMT1↓, FOXO1↑)	Reduces lipid droplet formation in renal tubular cells	Luo, et al. (2025)	Preliminary
Flavonoids	Calycosin	*In Vivo* and *In Vitro*	PA-induced HK-2 cells KKAy diabetic mice	PI3K/AKT Pathway (PI3K↑, AKT↑)	Reduces renal lipid accumulation	Zhang, et al. (2025b)	Strong

## 2 Lipid metabolic dysregulation in diabetic kidney disease

DKD is pathologically characterized by chronic hyperglycemia and profound lipid metabolic disturbances. Renal biopsies from DKD patients exhibited substantial lipid accumulation and increased intracellular lipid droplets compared to healthy controls (Herman-Edelstein et al., 2014). This lipid accumulation correlates with enhanced lipid synthesis and reduced catabolic processes in renal tubular and glomerular cells under hyperglycemic conditions, leading to aberrant fatty acid accumulation. Such metabolic derangements trigger inflammatory cascades and apoptotic pathways, ultimately compromising renal function (Tu et al., 2025; Zhang et al., 2025a). Lipotoxicity manifests through interconnected nephrotoxic mechanisms. First, cytotoxic lipid overload directly impairs cellular function, particularly glomerular integrity (DeFronzo et al., 2021). Studies reported significant lipid accumulation within glomeruli of obese and diabetic subjects, which accelerates glomerulosclerosis and tubular injury, thereby exacerbating renal dysfunction (Meléndez-Salcido et al., 2022). Second, lipid peroxidation amplifies oxidative stress by elevating reactive oxygen species (ROS) beyond endogenous antioxidant capacity. This oxidative environment disrupts cellular membranes, promotes protein denaturation, and induces renal dysfunction. Elevated ROS in diabetic kidneys originates primarily from excessive free fatty acids and lipid peroxides (Ankita et al., 2024). Furthermore, lipid peroxidation dysregulates renal cellular signaling pathways, impairing physiological cellular functions (Gago-Dominguez and Castelao, 2006; Tu et al., 2025). Lastly, lipid dysmetabolism activates localized inflammatory responses, releasing factors such as tumor necrosis factor-α (TNF-α) and interleukin-6 (IL-6), which not only aggravate glomerular and tubular damage but also promote kidney fibrosis (Fernández-Real et al., 2012; Zhang et al., 2012). Collectively, these pathomechanisms synergistically drive progressive structural and functional deterioration toward end-stage renal disease. Interventions targeting lipid dysregulation may mitigate DKD progression (Tu et al., 2025).

## 3 Mechanistic role of lipophagy in diabetic kidney disease

Lipophagy, a selective autophagic pathway targeting lipid droplets, preserves intracellular lipid homeostasis by orchestrating lysosomal degradation of neutral lipids. This process prevents lipotoxic cellular damage via coordinated actions of key molecular mediators. Autophagy related 5 (ATG5) serves as an essential component for autophagosome formation through its conjugation with ATG12, thereby facilitating autophagosomal biogenesis (Li and Wang, 2023; Wang et al., 2024). Microtubule-associated protein 1A/1B-light chain 3 (LC3) operates as a canonical autophagosomal membrane protein; the conversion of its cytosolic form (LC3-I) to phosphatidylethanolamine-conjugated form (LC3-II) signifies autophagic activity and mediates autophagosome maturation and lysosomal fusion (Li et al., 2023). The adaptor protein sequestosome 1 (SQSTM1/p62) acts as a pivotal scaffold linking lipid droplets to autophagic machinery, thereby promoting lipophagic degradation (Ruan et al., 2024). These molecular interactions regulate lipophagic flux and preserve cellular lipid homeostasis.

During the progression of DKD, lipophagic activity is impaired by oxidative stress, chronic inflammation, and metabolic imbalance, aggravating renal lipid accumulation and injury (Chae et al., 2023). Experimental investigations utilizing high-fat diet-induced murine models demonstrated significantly impaired lipophagy, evidenced by abnormal expression patterns of LC3-II and p62 proteins (Yamamoto et al., 2017). Mechanistically, lipid overload induces lysosomal dysfunction and impairs lipophagic flux in renal proximal tubular cells, intensifying renal lipotoxicity. The study indicated that renal cells in the diabetic environment initially activate lipophagy as a cytoprotective mechanism against lipid overload; however, once this pathway is compromised, cells experience severe metabolic stress, leading to functional impairment and accelerated renal pathology (Chae et al., 2023). Consequently, therapeutic strategies targeting molecular mediators of the lipophagy pathway, particularly those enhancing lipid droplet-autophagosome interactions, hold potential for DKD management.

## 4 Botanical medicine targets for lipophagy modulation

### 4.1 AMPK/mTOR signaling pathways

AMP-activated protein kinase (AMPK) serves as a cellular energy sensor activated during energy deficit to coordinate metabolic adaptations and maintain energy homeostasis. AMPK-mediated lipophagy promotion primarily occurs through suppression of downstream mechanistic target of rapamycin (mTOR) signaling (Chauhan et al., 2023). Under diabetic environment, AMPK activation enhances cellular fatty acid β-oxidation and stimulates autophagic clearance of lipids within renal tubular cells (Chauhan et al., 2023). Futhermore, mTOR acts as a pivotal signaling integrator that modulates cellular growth and metabolism in response to nutrient availability and growth factors (Goul et al., 2023; Shi et al., 2024). Upon AMPK activation, phosphorylation of downstream mTOR effectors suppresses mTOR activity, thereby inducing autophagy and enhancing cellular adaptation to energy stress (Oza et al., 2021). Importantly, mTOR hyperactivation potently inhibits autophagy, exacerbating intracellular lipid accumulation and renal cellular dysfunction (Oza et al., 2021). Collectively, AMPK/mTOR signaling represents a core regulatory axis for lipophagy modulation in DKD.

### 4.2 PPARα/γ signaling pathways

Peroxisome proliferator-activated receptors (PPARs), including isoforms PPARα, PPARβ/δ, and PPARγ, comprise ligand-activated nuclear transcription factors critically regulating lipid metabolism, inflammatory responses, and energy homeostasis (Chen et al., 2024). PPARα and PPARγ agonists demonstrate significant lipophagy-modulating capacities. Substantial evidence confirmed natural medicines ameliorate metabolic syndrome-related pathologies, including DKD and lipid dysregulation, via PPAR signaling modulation (Zou et al., 2023). Specifically, natural medicines such as resveratrol and genistein activate PPARα, subsequently enhancing fatty acid catabolism and mitigating ectopic lipid accumulation (Sharma et al., 2024). PPARγ agonists, including thiazolidinediones, clinically manage type 2 diabetes by improving insulin sensitivity, promoting adipocyte differentiation, and inhibiting adipocyte apoptosis (Ma et al., 2021; Sharma and Patial, 2022). These findings collectively underscore the centrality of PPAR signaling in lipid-metabolism regulation and highlight the effectiveness of natural medicines in improving lipid-metabolism disorders in DKD via PPAR-pathway activation.

### 4.3 SIRT1/FoxO1 signaling pathways

Sirtuin 1 (SIRT1), an NAD^+^-dependent deacetylase, orchestrates critical cellular processes including autophagy, metabolic regulation, and senescence. SIRT1 activates autophagic machinery through deacetylation, thereby enhancing intracellular lipid clearance. Mechanistically, SIRT1 activation deacetylates forkhead box protein O1 (FoxO1), augmenting FoxO1-mediated autophagic processes that promote lipid elimination (Wang et al., 2015; Xu M. et al., 2024). FoxO1, acting as a transcription factor, modulates expression of lipophagy-related genes such as adipose triglyceride lipase (ATGL), whose activation accelerates fatty acid oxidation and lipid hydrolysis (Chakrabarti et al., 2011; Zhang et al., 2016). Moreover, SIRT1 activation promotes autophagy via the AMPK signalling pathway, and its interaction with FoxO1 becomes particularly critical under diabetic conditions (Chang et al., 2015; Xu Y. et al., 2024). Therefore, pharmacologically enhancing SIRT1 activity to promote FoxO1 deacetylation may therapeutically ameliorate renal lipid dysmetabolism in DKD, offering novel biological targets and therapeutic rationale.

## 5 Natural medicines modulating renal lipophagy

### 5.1 Polyphenols

Polyphenols abundant in traditional medicinal and dietary plants orchestrate lipophagy chiefly via activation of central signaling cascades including AMPK, SIRT1 and transcription factor EB (TFEB) (Giulia et al., 2020; Zhenyu et al., 2023). Resveratrol, a non-flavonoid polyphenol derived from many dietary plants, ameliorates renal lipid accumulation by deacetylating FoxO3, peroxisome proliferator-activated receptor-γ coactivator-1α (PGC-1α), and Beclin1 (Claudia et al., 2022). In DKD models, Resveratrol significantly mitigates lipid accumulation and insulin resistance in DKD by activating autophagy via the AMPKα/mTOR pathway (Yong-Qi and Youjia, 2020). Specifically, it enhances phosphorylation of AMPKα, which inhibits mTOR activity and promotes autophagy activating kinase 1 (ULK1) activation. This cascade upregulates autophagy-related proteins Beclin1 and LC3 II/I, leading to the degradation of lipid droplets and improvement in renal lipid metabolism (Yong-Qi and Youjia, 2020). Beyond resveratrol, other polyphenolic metabolites exhibit nephroprotective efficacy. Ai et al. demonstrated that paeonol ameliorates DKD by activating TFEB-mediated lipophagy (Ai et al., 2025). Paeonol directly binds to RHEB, acting as an mTOR inhibitor, which promotes TFEB dephosphorylation and nuclear translocation. This activates the transcription of lysosomal genes lysosomal-associated membrane protein-2 (LAMP2), ATPase H + transporting V1 subunit A (ATP6V1A), and PGC-1α, enhancing lysosome biogenesis and restoring autophagic flux, ultimately leading to the clearance of excess renal tubular lipids and improved renal function (Ai et al., 2025).

### 5.2 Terpenoids

Commonly isolated from traditional medicinal plants, terpenoids exhibit significant effects on renal lipophagy. Celastrol, isolated from the botanical drug *Tripterygium wilfordii* Hook. f (Celastraceae), ameliorates DKD by activating renal lipophagy through PI3K/AKT/mTOR pathway inhibition. In high-fat/high-glucose-diet and STZ-induced diabetic rats, celastrol significantly downregulated PI3K, p-AKT, and mTOR expression, leading to increased LC3-II levels, thereby attenuating podocyte injury, glomerular basement membrane thickening, and proteinuria (Nie et al., 2020). In addition, celastrol exerts pronounced renal lipophagy-regulatory efficacy by inducing lipophagy in clear cell renal cell carcinoma via liver X receptor α (LXRα)/ATP-binding cassette transporter A1 (ABCA1) pathway activation (Chan-juan et al., 2020). Celastrol activates LXRα to transcriptionally upregulate ABCA1, which is essential for cholesterol efflux and autophagic degradation of lipid droplets. Concurrently, celastrol induces canonical autophagy by inhibiting mTOR activity, enhancing LC3-I-to-LC3-II conversion, and degrading p62, thereby promoting lipid droplet sequestration and lysosomal breakdown (Chan-juan et al., 2020). By alleviating this lipid-rich environment, celastrol inhibits the epithelial-mesenchymal transition process, ultimately suppressing cancer cell proliferation, migration, invasion, and tumor growth (Chan-juan et al., 2020). Other terpenoids also contribute to renal therapeutics. Morroniside, a secoiridoid metabolite from *Cornus officinalis* Siebold and Zucc (Cornaceae), attenuates lipid metabolism disorder-driven chronic kidney disease by activating AMPKα/TFEB-mediated lipophagy (Zhang C. et al., 2025). Specifically, morroniside promotes AMPKα phosphorylation, triggering TFEB nuclear translocation to upregulate lipophagy, thereby enhancing ectopic lipid droplet clearance in high-fat/high-fructose diet-fed mice and palmitic acid-injured renal tubular cells (Zhang C. et al., 2025). Geniposide, an iridoid glycoside from *Gardenia jasminoides* J. Ellis (Rubiaceae), exerts renoprotective effects in DKD primarily by enhancing autophagy and reducing oxidative stress. It activates the AMPK/ULK1 autophagy pathway through phosphorylation of AMPK and ULK1, while simultaneously inhibiting AKT phosphorylation, reducing 4-HNE accumulation, TUNEL-positive apoptotic cells, and elevating NAD(P) H quinone oxidoreductase-1 (NQO-1), manganese superoxide dismutase (MnSOD2) and glutathione peroxidase-1 (GPX-1) expression (Theodomir et al., 2021).

### 5.3 Alkaloids

Characteristically obtained from medicinal plants, alkaloids like berberine reshape lipid droplet homeostasis by modulating SIRT3-mediated lipophagy, partially alleviating cardiac lipotoxicity in diabetic cardiomyopathy. Given the pathophysiological parallels between diabetic cardiomyopathy and DKD, including lipid accumulation and lipotoxicity, berberine, an isoquinoline alkaloid from *Coptis chinensis* Franch (Ranunculaceae), may confer analogous lipophagic benefits in DKD (Chen et al., 2025). Furthermore, berberine inhibits the mTOR/ribosomal protein S6 kinase 1 (P70S6K)/eukaryotic translation initiation factor 4E-binding protein 1 (4EBP1) signaling axis to activate podocyte autophagy, thereby attenuating high glucose-induced podocyte apoptosis (Li et al., 2020). In induced acute kidney injury (AKI) model, berberine conferred renoprotection by regulating the histone deacetylase 4 (HDAC4)-FoxO3a axis to induce autophagy and inhibit apoptosis (Zhi et al., 2023). Berberine also upregulates Klotho expression in cisplatin-induced AKI, activating the AMPK/mTOR/ULK1/Beclin-1 autophagy pathway to mitigate oxidative injury (SOD activity increased by 86%; MDA decreased by 54%), inflammation, and cell death (Salah et al., 2025). Additionally, neferine, a bisbenzylisoquinoline alkaloid from *Nelumbo nucifera* Gaertn (Nelumbonaceae), protects against cisplatin-induced AKI by activating autophagy via the AMPK/mTOR pathway, significantly reducing renal injury (Li et al., 2023). Although lipid autophagy was not explicitly examined, neferine’s AMPK-driven autophagy induction aligns with mechanisms governing lipophagy, suggesting broad applicability in lipid clearance during renal injury.

### 5.4 Polysaccharides

Primarily extracted from diverse natural sources such as algae, polysaccharides demonstrate notable renoprotective properties. Trehalose, a non-reducing disaccharide isolated from *Selaginella lepidophylla* (Hook. and Grev.) Spring (Selaginellaceae), demonstrates notable renoprotective properties. Studies indicated that it alleviates renal ischemia-reperfusion injury, potentially via autophagy enhancement coupled with oxidative stress, inflammation, and apoptosis suppression (Lingling et al., 2020; Suwen et al., 2020). Specifically, Trehalose’s renoprotective effects are primarily mediated through TFEB-driven autophagy, which indirectly mitigates renal damage by clearing oxidized organelles and suppressing oxidative stress (Lingling et al., 2020; Suwen, et al., 2020). Fucoidan, a principal constituent of the Chinese medicinal preparation Haikun Shenxi, attenuates renal cellular senescence phenotypes, including SA-β-galactosidase activity and klotho/p53/p21 expression by modulating autophagy-associated AMPK/ULK1 signaling (Qian et al., 2020). Although studies on trehalose and fucoidan have not explicitly addressed lipophagy, they demonstrated that trehalose enhances global autophagy, which in turn indirectly influences lipid metabolism.

### 5.5 Flavonoids

Flavonoids, derived from fruits, vegetables, and medicinal plants, have garnered considerable scientific interest owing to their antioxidant, anti-inflammatory, and antidiabetic properties, demonstrating remarkable efficacy in countering lipid dysregulation associated with DKD (Hu et al., 2021). Diosgenin, extracted from *Dioscorea zingiberensis* C.H.Wright (Dioscoreaceae), has garnered attention for its beneficial effects on lipid metabolism in DKD, improving lipid metabolism by regulating the miR-148b-3p/DNA methyltransferase 1 (DNMT1)/FOXO1 axis and inhibiting lipid droplet formation in human kidney-2 (HK-2) cells (Luo et al., 2025). Calycosin, an isoflavonoid metabolite from *Astragalus mongholicus* Bunge (Fabaceae), mitigates lipid accumulation in DKD by restoring autophagy via phosphatidylinositol 3-kinase (PI3K)/protein kinase B (AKT) pathway inhibition (Zhang et al., 2025b). Marein, derived from *Coreopsis tinctoria* Nutt (Asteraceae), mitigated lipid accumulation by inducing lipophagy through suppression of the PI3K/AKT/mTOR pathway (Zhang et al., 2023). In sodium oleate-induced hepatoma G2 (HepG2) cells and High fat and sugar diet (HFSD)-fed mice, Marein downregulates p-PI3K, p-AKT, and p-mTOR, leading to increased LC3-II/LC3-I ratios and enhanced co-localization of autophagosomes with lipid droplets. This promotes lysosomal degradation of lipids, reducing cellular triglycerides (TG), total cholesterol (TC), and low-density lipoprotein cholesterol (LDL-C) while elevating high-density lipoprotein cholesterol (HDL-C) (Zhang et al., 2023). Notably, marein promotes renal autophagy in DKD by activating the PI3K/AKT pathway while inhibiting mTOR, evidenced by upregulated LC3-II/I, Beclin-1 and ATG5 with p62 degradation, thereby improving insulin sensitivity and attenuating nephropathy in db/db mice (Li et al., 2021). Quercetin, a flavonoid abundantly present in fruits and vegetables, has been substantiated to exert beneficial regulatory effects on autophagic processes. In the context of DKD, quercetin alleviates renal fibrosis by inhibiting excessive AMPK-dependent autophagy (Lai et al., 2020). In high-fat diet/streptozotocin-induced type 2 diabetic rats and high glucose-treated rat mesangial cells, quercetin downregulated key autophagy markers, including LC3II/I, Beclin-1, ATG5, and increased P62 accumulation in kidney tissue, concurrently reducing collagen deposition (Lai et al., 2020). Conversely, In the context of metabolic dysfunction-associated steatotic liver disease (MASLD), quercetin inhibits the mTOR/Yin-Yang1 (YY1) pathway, relieving its suppression on lipophagy and enhancing autophagic flux and lipid droplet degradation, as evidenced by increased Beclin1 expression and LC3-II conversion alongside decreased p62 levels (Katsaros et al., 2024). This divergence reflects a cell-type and disease-stage-specific effect: hepatocytes under lipid overload benefit from autophagy activation, whereas rat mesangial cells exposed to chronic hyperglycemia are adversely affected by autophagy overactivation. In addition, quercetin should be regarded neither as a universal autophagy activator nor as an inhibitor. Its net effect is dictated by tissue-specific signalling networks and dosing regimens. Thus, these contrasting outcomes underscore the necessity of conducting tissue-specific dose–response studies prior to translating quercetin or any other autophagy-modulating agent into human trials for DKD.

Cross-class comparison reveals that AMPK/mTOR modulation is the most consistently reported mechanism, being independently documented for resveratrol, celastrol, geniposide, berberine and marein in at least two different DKD models each. In contrast, TFEB-mediated lysosome biogenesis has been verified only for paeonol and morroniside, while SIRT3-dependent lipophagy is presently limited to berberine in diabetic cardiomyopathy and warrants confirmation in DKD models. Simultaneous engagement of AMPK/mTOR and SIRT1/FoxO3 axes by resveratrol suggests potential synergy, whereas PI3K/AKT inhibition is confined to celastrol, calycosin and marein. This convergence indicates that distinct natural medicines have independently evolved to target the same nutrient-sensing and autophagy checkpoints. Notably, Several of the polyphenols and flavonoids discussed above have been classified as pan-assay interfering substances (PAINS) that can produce false-positive read-outs in biochemical or cell-free screens owing to metal chelation, redox reactivity, aggrega-tion, or membrane modulation (Magalhães et al., 2021). Future work should incorporate PAINS-aware chemical filters, counter-screens for aggregation and, whenever possible, orthogonal genetic or clinical trial validation.

## 6 Clinical-translational outlook

The clinical translation of natural medicines that promote lipophagic activity represents an intricate and formidable endeavor. Although the role of natural medicines in modulating lipophagy has been extensively investigated, their transition into clinical application continues to confront considerable challenges. First and foremost, the safety of natural medicines is a critical issue in clinical translation. A study has revealed that berberine induces significant cardiotoxicity *in vitro* and complete cardiac arrest at concentrations reaching 10 μM (Zhang et al., 2018). Furthermore, a 26-week chronic oral toxicity investigation in rats identified marked hepatorenal toxicity following geniposide administration at a dosage of 100 mg/kg/day (Tian et al., 2018). Additionally, the potential for pharmacokinetic interactions upon co-administration with conventional therapeutics presents a substantial complicating factor. A study indicated that resveratrol and kaempferol inhibit aryl hydrocarbon receptor (AHR)-mediated transcription, potentially modulating cytochrome P450 enzymes such as CYP1A1 and CYP1B1. This interference may disrupt the metabolic pathways of concomitant medications, including anthracycline-based chemotherapeutic agents (MacPherson and Matthews, 2010). Hence, systematic investigation into the pharmacological and toxicological properties of natural agents, alongside their interactive potential with modern pharmaceuticals, is indispensable for ensuring both safety and therapeutic efficacy. It is also pertinent to note that the scarcity of robust clinical trial evidence constitutes a significant impediment to widespread clinical adoption. A randomized, double-blind, placebo-controlled study evaluated the efficacy of resveratrol in sixty patients presenting with type 2 diabetes and proteinuria (Sattarinezhad et al., 2019). The study found that resveratrol significantly reduced the urinary albumin-to-creatinine ratio, although no significant changes were observed in glomerular filtration rate or serum creatinine, and it enhanced serum antioxidant enzyme levels (Sattarinezhad et al., 2019). Clinical data pertaining to other metabolites that induce lipophagy remain exceedingly sparse, despite a proliferation of *in vitro* and *in vivo* studies corroborating their biological activities. Finally, study employing animal and cellular models is inherently constrained by the limitations of these systems. Although existing rodent models emulate certain features of DKD, such as albuminuria and glomerular matrix expansion, none comprehensively replicate the entirety of human disease pathology, particularly advanced lesions including nodular glomerulosclerosis and progressive renal failure (Kitada et al., 2016). Moreover, variables such as genetic background, strain-specific susceptibilities, comorbid conditions (e.g., hypertension, IgA deposition), and methodologies of model induction (e.g., streptozotocin toxicity, lipotoxicity) further constrain the translatability of findings derived from these systems (Kitada et al., 2016). Cellular models, while illuminating molecular mechanisms, often exhibit substantial discrepancies between *in vitro* behavior and *in vivo* physiological responses. These inherent limitations collectively impede the clinical translation of natural medicines. Thus, the development of more sophisticated animal models or the adoption of advanced *in vitro* systems, such as three-dimensional cell cultures and organ-on-a-chip technologies, capable of more faithfully mimicking human pathophysiology, would markedly enhance translational efficiency.

## 7 Conclusion

The therapeutic potential of natural medicines in DKD is increasingly recognized, particularly for their distinct advantages in ameliorating lipid metabolic disorders via lipophagic modulation. Substantial evidence confirms that natural medicines, including flavonoids, polyphenols, terpenoids, alkaloids, and polysaccharides, orchestrate lipophagy through multiple signaling pathways, thereby reducing renal lipid deposition and oxidative stress while promoting pathological amelioration in DKD. Despite encouraging findings, the precise molecular mechanisms underlying natural medicine-mediated lipophagic regulation warrant deeper exploration. While natural medicines present a promising frontier for DKD research and application, formidable obstacles remain. Future studies should prioritize the precise identification of natural medicines, systematic validation of their targets, rigorous assessment of clinical efficacy, translatability, and safety profiles.
